# Prevalence and predictors of adolescents’ cigarette smoking in Madinah, Saudi Arabia: a school-based cross-sectional study

**DOI:** 10.1186/s12889-015-1363-8

**Published:** 2015-01-21

**Authors:** Abdulmohsen Al-Zalabani, Khaled Kasim

**Affiliations:** Family and Community Medicine Department, College of Medicine, Taibah University, Madinah, KSA 30001 Saudi Arabia; Medical Education Department, College of Medicine, Taibah University, Madinah, KSA 30001 Saudi Arabia; Public Health and Community Medicine Department, Faculty of Medicine, Al-Azhar University, Cairo, Egypt

**Keywords:** Adolescents, Prevalence, Risk factors, Smoking, Saudi Arabia

## Abstract

**Background:**

Although the prevalence of adolescents’ cigarette smoking has increased in recent decades, little is known regarding its epidemiology in certain Saudi regions, including the Madinah region. The aim of this study was to determine the prevalence and predictors of adolescent cigarette smoking in Madinah, Saudi Arabia.

**Methods:**

A school-based cross-sectional study was carried out in the Madinah region during 2013. A multistage stratified cluster sample was taken and included 3400 students (11–19 years) from 34 intermediate and secondary schools. Data concerning sociodemographic and smoking-related factors were collected using a valid and reliable self-administered questionnaire. The prevalence of smoking was estimated, and appropriate statistical analyses were performed, including univariate, predictive and multivariate regression analyses.

**Results:**

The overall response rate was 97.7%. The prevalence of cigarette smoking in the respondents’ 3322 adolescents was 15.17% (95% CI = 13.95-16.39) with significant differences in sociodemographic factors. The most important predictors were most or all friends smoking (OR = 12.5; 95% CI = 9.40-16.8). Other significant less important factors were parental smoking, belief in the harmful effects of smoking, cigarette advertisement in mass media, and pocket money.

**Conclusions:**

Cigarette smoking prevalence is a relatively low among adolescents in Madinah region. However, friends and parents smoking play an important role in the increased risk of smoking in the studied adolescents. These predictors must be included in any antismoking education programs targeting to this sector of population in the region.

## Background

Cigarette smoking and its health consequences represents one of the most serious public health problems and represents an important health challenge worldwide [1]. Evidence is accumulating that smoking increases the risk of nearly all types of cancers and cardiovascular diseases [2]. According to the World Health Organization, tobacco use causes one in ten deaths among adults and represents the main cause of premature death globally [1]. In the last two decades, adolescents have become more exposed to tobacco promotion and marketing at early ages [3], and most smokers have reported that they began smoking before age 18 [4-6].

Several risk factors have been implicated in the prediction of adolescent smoking. These factors include older age, male sex [7], parental smoking [8,9], friends smoking [10,11], socioeconomic status [10,12], family problems [13], perception that smoking was harmful to health [14], and tobacco advertisements [14,15].

Among the population aged 15 or more in Saudi Arabia, approximately 37.6% of males and 6% of females are current smokers [3]. In 2009 article, Bassiony reviewed all published papers that investigated tobacco use and cigarette smoking among secondary school and university students in Saudi Arabia between 1987 and 2008. There were 22 studies; 10 included secondary school students, and 12 included university students. Most of these studies were conducted in Riyadh, Saudi Arabia, and no study from Madinah was found. According to this review, the prevalence of cigarette smoking in secondary school students ranged from 12% to 29.8%, and among university students, it ranged from 2.4% to 37%, with the lowest prevalence being among female and medical students [16]. A similar high prevalence of adolescents’ cigarette smoking was also reported in recent Saudi studies conducted in Jeddah [11] and Riyadh [17,18]. The prevalence rate of adolescent smoking in these studies was 37%, 31% and 29%, respectively.

Despite the above-mentioned studies, including those in the Bassiony review, there is still a lack in Saudi literature about adolescent smoking and its predictors in certain Saudi Arabia regions, including the Madinah city. From this point of view, the present study aimed to estimate the current prevalence of cigarette smoking among intermediate and secondary school students in the Madinah region and to determine the most important predictors associated with the risk of adolescent smoking in this region.

## Methods

The present school-based cross sectional study recruited intermediate and secondary school students from Madinah city, Saudi Arabia, to determine the prevalence and predictors of cigarette smoking among them. Madinah city is the second holiest city in the Hejaz region of western Saudi Arabia, and the capital of Al Madinah Province. The Saudi population of Madinah city was 784,722 according to 2010 Census [19]. The basic educational system in Saudi Arabia is divided into primary, intermediate and secondary. Primary education lasts six years, and children at the age of 6 enter the first grade of primary education. Intermediate education lasts three years, and secondary education lasts three years and this is the final stage of general education [20]. In this study, all intermediate and secondary school students in Madinah city were eligible, and no exclusion criteria were used.

### Sampling procedures

The study analyzed data from 3322 intermediate and secondary school students from the Madinah region, Saudi Arabia, to determine the prevalence and predictors of cigarette smoking among them. A multistage, stratified cluster sampling procedure was used. The schools in the Madinah region have been defined in strata according to level (intermediate vs. secondary), status (public vs. private) and students’ sex (male vs. female), with the final sample being proportional to the size of the stratum. Within each stratum, a cluster sampling was used in which the primary sampling unit was the school. Schools were selected in proportion to their size. Finally, from each randomly selected school, one class from each grade was randomly selected. All students in each selected class were invited to participate.

The primary calculated sample of this study was 780 students based on averages of the estimated smoking prevalence among school students in previous Saudi studies (20-30%), an assumed precision of 3% and a confidence interval of 95%. To obtain the same level of accuracy in both male and female students as well as in intermediate and secondary schools, the sample size was quadrupled to 3120 students. Accounting for non-response, the sample size increases to 3400 students.

### Data collection

The questionnaire used in this study was adapted from the Global Youth Tobacco Survey (GYTS) questionnaire [19]. The GYTS is a self-administered questionnaire consisting of 56 core questions designed to gather data on the prevalence of cigarette smoking and its associated risk factors. The questionnaire covered data about the sociodemographic and behavioral characteristics of the respondents, and included questions about their smoking status, smoking related factors, and knowledge about the hazards and long-term consequences of smoking.

All question items were translated into Arabic, which was verified by back-translation performed by a different bilingual person. Disagreement in the translation was resolved by discussion between translators and the research team. The validity of the Arabic questionnaire used was obtained from discussions with public health and tobacco-control experts, and the estimated reliability exceeded 98%.

The students were interviewed by trained public health personnel and asked to fill the Arabic version of the used GYTS questionnaire. The school officials were clearly informed about the aim and scope of the study. No student was obliged to participate in the study. Before the administration of the study questionnaire, the interviewer read out the consent form to obtain written consent of the participants and/or their teachers as Taibah University Ethics Committee waived the need for parental consent for those participants younger than 16 years. Ethical consideration was also taken to ensure the confidentiality and privacy of the collected data. Finally, the Deanship of Scientific Research Ethics Committee at Taibah University, Madinah, Saudi Arabia, approved the study protocol.

### Study variables

Smoking status, the dependent variable, was assessed in the study questionnaire by the following questions: “During the past 30 days, how many days did you smoke cigarettes?”, followed by, “During the past 30 days, on the days you smoke, how many cigarettes did you usually smoke?”, and, “How old were you when you first tried a cigarette?”. The current smokers were defined as students who had smoked at least once in the past 30 days while never smokers were those who had never tried smoking in their lifetime [21].

The explanatory variables in this study were: i) Sociodemographic characteristics: age in years (≤13, 14, 15, ≥ 16), sex (male vs. female), school level (intermediate vs. secondary), school status (public vs. private), pocket money (≤300SR vs. > 300 SR), education of mother and father (illiterate, basic education, and university or higher); ii) Smoking related factors: parents smoking (none, one parent smokes, both parents smoke), best friends smoking (none, some, most or all); belief that smoking is harmful (yes vs. no), belief that smokers have more friends (yes vs. no); and iii) Having seen cigarette advertisements in mass media (yes vs. no).

### Statistical analysis

All data analyses were performed using the Statistical Analysis System software package [22]. Descriptive statistics were used to estimate smoking prevalence. The chi square test was used to compare the smoking status of the studied adolescents by their sociodemographic characteristics. The level of statistical significance was defined as *P* ≤ 0.05. The regression analyses were first performed, variable-by-variable (univariate analyses) to estimate odds ratios (OR) and their 95% confidence intervals (95% CI) for the association of adolescents smoking with the studied risk factors. Then, all variables with a statistically significant association with the dependent variable (smoking) were included in the final predictive model based on the stepwise regression with a p value of 0.01 as entry criterion and a p value of 0.05 as exclusion criterion. Finally, the obtained predictors from stepwise model were entered into multivariate logistic regression model while controlling for age, sex, school level and parents' education as possible known confounders.

## Results

The overall response rate in this study was 97.7% (3322/3400), with no significant school level difference. The response rate was 97.5% (1561/1600) in intermediate schools, and 97.8% (1761/1800) in secondary schools. The prevalence of smoking among the studied adolescents was 15.17% (504/3322 (95% CI = 13.95-16.39)). The majority of adolescent smokers in this sample (75%) reported starting smoking before the age of 14 with no significant sex difference. Among smokers, those who reported to smoke more than 10 cigarettes per day were about 16%.

Table 1 presents the prevalence of smoking among adolescents by their studied sociodemographic factors. There were statistically significant differences of adolescent smoking regarding all studied factors. The prevalence of smoking was higher among those aged 16 or more (18.3%), male (21.3%), secondary school students (16.3%), private school students (17.4%), adolescents reported pocket money less than 300 SR (14.3.0%), and adolescents in the illiterate father (24.2%) and mother (24.0%) groups.

**Table 1:** **Current prevalence of adolescents' cigarette smoking by their sociodemographic characteristics in Madinah, Saudi Arabia**

**Characteristics**	**Smokers (n = 504)**		**Non-smokers (n = 2818)**		**P value**
	No.	%*	No.	%*	
**Age group in years**					
≤13	65	12.4	461	87.6	<.0001**
14-	45	9.4	434	90.6	
15-	77	13.3	506	86.2	
≥16	317	18.3	1417	81.5	
**Sex**					
Female	129	8.3	1430	91.7	<.0001**
Male	375	21.3	1388	78.7	
**School level**					
Intermediate	217	13.9	1344	86.1	0.04**
Secondary	287	16.3	1474	83.7	
**School status**					
Public	438	14.9	2504	85.1	0.20
Private	66	17.4	314	82.6	
**Pocket money per month**					
≤300 SR	425	14.3	2558	85.6	0.001**
>300 SR	79	23.3	260	76.7	
**Father education**					
Illiterate	62	24.2	194	75.8	0.0001**
Less than university	277	15.0	1573	85.0	
University and higher	165	13.5	1051	86.5	
**Mother education**					
Illiterate	76	24.0	240	76.0	0.001**
Less than university	276	13.3	1794	86.7	
University and higher	152	16.2	784	83.8	

*Percentages are rounded.

**Significant.

Table 2 showed the risk of adolescents' smoking with sociodemographic factors in the univariate regression models. The risk of smoking is found to increase with increasing age of the adolescents, and a significant positive association was detected among those aged 16 or more (OR = 1.17; 95% CI = 1.01-1.28). Sex of the adolescent, school level, school status, and pocket money were also shown to be positive associations with smoking. The highest positive and significant association was among male sex (OR = 3.00; 95% CI = 2.42-3.70), and monthly pocket money more than 300SR (OR = 1.83; 95% CI = 1.39-2.40). Parents’ education, however, appeared to have reducing effects on the risk of adolescents smoking. The risk was reduced by approximately 51% and 39%, respectively, among adolescents in highly educated father and mother groups.

**Table 2:** **Association of sociodemographic factors with the risk of adolescents’ cigarette smoking in Madinah, Saudi Arabia**

	**Smokers (n = 504)**	**Non-smokers (n = 2818)**	**OR**	**95% CI**
**Age group in years**				
≤13	65	461	1.00	Ref.
14-	45	434	0.74	0.49-1.10
15-	77	506	1.04	0.87-1.24
≥16	317	1417	1.17	1.06-1.28
**Sex**				
Female	129	1430	1.00	Ref.
Male	375	1388	3.00	2.40-3.70
**School level**				
Intermediate	217	1344	1.00	Ref.
Secondary	287	1474	1.21	0.99-1.46
**School status**				
Public	438	2504	1.00	Ref.
Private	66	314	1.20	0.90-1.60
**Pocket money per month**				
≤300 SR	425	2558	1.00	Ref.
>300 SR	79	260	1.83	1.39-2.40
**Father education**				
Illiterate	62	194	1.00	Ref.
Less than university	277	1573	0.55	0.40-0.75
University and higher	165	1051	0.49	0.35-0.68
**Mother education**				
Illiterate	76	240	1.00	Ref.
Less than university	276	1794	0.49	0.36-0.65
University and higher	152	784	0.61	0.44-0.84

Table 3 shows the univariate analyses for the association between adolescents' smoking and smoking-related factors. There has been a significant positive association between smoking and the following factors: parents smoking, friends smoking, and cigarette advertisements. The highly significant risks were associated with friends smoking in terms of most or all friends smoking (OR = 14.10; 95% CI = 10.60-18.8), and some friends smoking (4.70; 95% CI = 4.43-7.36). Parental smoking was also positively associated with adolescent smoking. The risk was 1.69 (95% CI = 1.35-2.10) among adolescents having one parent smoking and 4.50 (95% CI = 2.70-7.50) among those having both parents smoking. The risk was also increased among adolescents who reported seeing cigarette advertisements in different mass media (OR = 1.50; 95% CI = 1.19-1.87). In contrast, however, the risk of smoking was reduced among adolescents who believed in the harmful effects of smoking (OR = 0.40; 95% CI = 0.32-0.53).

**Table 3:** **Association of smoking related factors with the risk of adolescents’ cigarette smoking in Madinah, Saudi Arabia**

	**Smokers (n = 504)**	**Non-smoker (n = 2818)**	**OR**	**95% CI**
**Parents smoking**				
No	393	2254	1.00	Ref.
One parent	135	526	1.69	1.35-2.10
Both	26	38	4.50	2.70-7.50
**Friends smoking**				
No	94	1799	1.00	Ref.
Some	233	780	4.70	4.43-7.36
Most or all	127	239	14.10	10.6-18.8
**Belief that smoking is harmful**				
No	96	247	1.00	Ref.
Yes	408	2571	0.40	0.32-0.53
**Belief that smokers have more friends**				
No	196	962	1.00	Ref.
Yes	308	1856	1.22	1.00-1.49
**Have seen cigarette advertisements in mass media**				
No	499	2319	1.00	Ref.
Yes	123	381	1.50	1.19-1.87

Table 4 displays the results of the predictive regression model including all variables with statistically significant odds ratios in the previous tables. The adjusted predictors of adolescents' smoking in this study were friends smoking, parents smoking, belief in the harmful effects of smoking, and cigarette advertisement in mass media. The risk was significantly increased among adolescents having most or all friends smoking (OR = 12.5; 95% CI = 9.40-16.8)., some friends smoking (5.70; 95% CI = 4.40-7.37), both parents smoking (2.95; 95% CI = 1.60-5.22), those reported monthly pocket money more than 300SR (OR = 1.65; 95% CI = 1.22-2.23), and among those reported seeing cigarette advertisements in different mass media (OR = 1.25; 95% CI = 0.95-1.55). According to this predictive model, however, the risk was significantly reduced by 60% among adolescents who believed in the harmful effects of smoking (OR = 0.40; 95% CI = 0.30-0.55).

**Table 4:** Predictors of adolescents' cigarette smoking in Madinah, Saudi Arabia*

	**Smokers (n = 504)**	**Non-smokers (n = 2818)**	OR**	**95% CI**
**Friends smoking**				
No	94	1799	1.00	Ref.
Some	233	780	5.70	4.40-7.37
Most or all	127	239	12.5	9.40-16.8
**Parents smoking**				
No	393	2254	1.00	Ref.
One parent	135	526	1.30	1.01-1.65
Both	26	38	2.95	1.60-5.22
**Pocket money per month**				
≤300 SR	425	2558	1.00	Ref.
>300 SR	79	260	1.65	1.22-2.23
**Have seen cigarette advertisements in mass media**				
No	499	2319	1.00	Ref.
Yes	123	381	1.25	0.95-1.55
**Belief that smoking is harmful**				
No	96	247	1.00	Ref.
Yes	408	2571	0.40	0.30-0.55

^*^Based on predictor regression model; including variables being significant in Tables 2 and 3.

**OR adjusted by age, sex, school level and parents’ education.

## Discussion

The prevalence of adolescents smoking in Madinah, Saudi Arabia, was 15.17%. The prevalence was significantly higher among male (21.3%) and secondary school students (16.3%). Further analyses by sex and school level showed a significantly high prevalence of smoking among male secondary school students compared to female students (26.2% vs. 7.9%), and among male intermediate school students compared to female (17.1% vs 8.8%). A much higher prevalence of smoking among male secondary school students was also reported in recent Saudi studies. One study included 85 secondary schools for males in Jeddah, Saudi Arabia, reported a prevalence of 37.1% among the studied male adolescents [11]. In the Riyadh region, a smoking prevalence of 31.2% among male secondary school students age 16–18 was reported [17]. In Al-Qassim and Tabouk regions, the prevalence of smoking among secondary school boys was also high and it was 29.8% and 34%, respectively [23,24]. The lower rate of adolescent smoking in Madinah compared to the above mentioned cities may be attributed to the fact that; in 2002, the two holy cities of Macca and Madinah were declared cigarette free by the Custodian of the Two Holy Masjids. Since then, tremendous efforts are being exerted by the Tobacco Control Program, Ministry of Health and the Anti-Smoking Committee (nongovernmental organization) towards maintaining and ensuring the continuity of this initiative [25].

The prevalence of cigarette smoking in the southern region of Saudi Arabia (Jazan city), however, was as low as 12.8% and 7.9% among male secondary and male intermediate school students [10]; and 3.8% among all studied female students. The lower rate observed in the Jazan city, however, could be attributed to the mode of living in this region, which is completely different from that in more urbanized Saudi regions. The mode of life in this city, Jazan, imposes more supervision on teenagers because the people live in tribal links with their extended families and relatives [10].

The positive association observed in this study between adolescent smoking and age, sex, school level, school status, and pocket money reinforces the role of sociodemographic characteristics in adolescent smoking reported in other studies [26-28].

The results of both univariate and predictive regression models have revealed the great role friends and parents smoking plays in the increased risk of smoking in the studied adolescents. In the predictive model, the risk was significantly increased 12.5-fold among adolescents who reported most or all friends smoking and 5.7-fold among those who reported some friends smoking. The risk was also increased 1.3 times among adolescents having one parent smoking and 2.95 times among those having both parents smoking. These findings appeared consistent with that reported in recent local [10,11,17,18] and international studies [13-15]. It is not surprising that parents and friends smoking were the most powerful predictors of adolescents smoking in this and other studies because most adolescents look up to family members and peers in their community and try to imitate their behavior. Furthermore, adolescents at this age tend to make friendships and social networks with their peers. The friendship pressure is expected to play an important role in the adolescents’ behavior toward smoking, especially when combined with a lack of parental support and supervision, family problems [13], and the presence of a smoking household member [29].

Belief that smoking is harmful to health was found to be negatively associated with the risk of adolescent smoking. The risk was significantly reduced by 40% in both univariate and predictive models. Similar risk reduction was also obtained in previous studies conducted in the Republic of Congo [14] and in Greece [15], where the perception of the hazards and long-term consequences of smoking was negatively associated with adolescents smoking in these countries. These findings may reflect the crucial role of antismoking messages in schools, clubs, and other youth meeting places in discouraging cigarette use among adolescents.

In both univariate and predictive models, cigarette advertisements in mass media, such as TV and newspapers/magazines appeared to have a positive effect on the risk of adolescent smoking in this study. The risk of smoking was significantly increased among adolescents who reported that they have seen cigarette advertisements in the univariate model with OR of 1.50 (95% CI = 1.19-1.87). In a previous study, the risk was 1.9 times higher among such adolescents exposed to cigarette advertisements in mass media [14]. In another study, anti-tobacco advertisements, like health warning messages on cigarette packs, have been associated with reduced adolescents smoking risk [30].

The negative association between parents’ education and adolescents smoking was only observed in the univariate model. The risk of smoking was reduced by 51% and 39% among adolescents in highly educated father and mother groups. Additionally, the prevalence of smoking was significantly higher among those adolescents in the illiterate father (24.2%) and mother (24%) group compared to those in the basic-education and highly educated parental groups. The educational level of parents is known to have a significant effect on adolescents’ values and beliefs towards smoking and may lower the rate of developing a smoking habit among them [31].

The present study appeared to have a number of strengths, include being a school-based study with a relatively large sample size and high response rate, which consolidate the study findings. The study questionnaire was comprehensive, being based on the GYTS questionnaire, and included most of the possible risk factors associated with adolescent smoking. To the best of our knowledge, this study is the first to examine the risk of adolescent smoking associated with a relatively large number of sociodemographic and smoking-related variables in Madinah. Finally, all estimated risks in this study have shown a high precision, as indicated by the observed narrowness of their confidence intervals.

This study has also number of limitations. The validation of self-reports via biomedical tests was not conducted because of organizational, logistic, and cultural constraints. This could underestimate the actual prevalence of smoking among the studied adolescents. The previous finding [32], however, suggests that an effective procedure to ensure anonymity can reduce the potential pressure among young people not to report their smoking habits truthfully, so the biochemical assessment of smoking is not essential. In this study, the questionnaires were self-administered anonymously. Because of the cross sectional nature, the causal influences of the risk factors cannot be determined in this study. However, the consistent strength and significant results obtained in this and other similar studies endorse both the role of these factors in adolescents smoking and the need to address them in any adolescent smoking prevention programs tailored to youth and school students.

## Conclusions

The prevalence of adolescent smoking in Madinah, Saudi Arabia is a relatively low. Friends smoking, parents smoking, cigarette advertisement in mass media and monthly pocket money were the most important predictors increasing the risk of smoking in the studied adolescents. Belief in the harmful health effects of smoking, however, was associated with a reduced risk of adolescent smoking. These findings reflect the need to design an appropriate and effective antismoking education program addressing these predictors and targeting not only school students but also their friends, families and school members.
